# Long-Term Feasibility of 13.56 MHz Modulated Electro-Hyperthermia-Based Preoperative Thermoradiochemotherapy in Locally Advanced Rectal Cancer

**DOI:** 10.3390/cancers14051271

**Published:** 2022-03-01

**Authors:** Yohan Lee, Sunghyun Kim, Hyejung Cha, Jae Hun Han, Hyun Joon Choi, Eun Go, Sei Hwan You

**Affiliations:** 1 Department of Radiation Oncology, Wonju Severance Christian Hospital, Yonsei University Wonju College of Medicine, Wonju 26426, Korea; 2030john3636@gmail.com (Y.L.); tjdgus9410@naver.com (S.K.); hyejungcha@yonsei.ac.kr (H.C.); hjchoi1@yonsei.ac.kr (H.J.C.); 2 Department of Biostatistics, Yonsei University Wonju College of Medicine, Wonju 26426, Korea; cpflhan@yonsei.ac.kr; 3 Department of Software, College of Software and Digital Healthcare Convergence, Yonsei University, Wonju 26493, Korea; 4him1@naver.com; 4 Center of Evidence-Based Medicine, Institute of Convergence Science, Yonsei University, Seoul 03722, Korea

**Keywords:** regional hyperthermia, rectal cancer, neoadjuvant chemoradiation, survival

## Abstract

**Simple Summary:**

We demonstrated that a 13.56 MHz modulated electro-hyperthermia (mEHT) boost is feasible in neoadjuvant treatment for rectal cancer. Herein, we attempted to present the long-term results for this phase 2 trial. Although there are many reports on the usefulness of thermoradiochemotherapy for loco-regional control, so far, only a few cases of survival benefit exist. Thus, this study assessed whether this limitation of hyperthermia could be overcome through the mEHT method featuring an applied energy variable. Following a median follow-up of 58 months for 60 patients, mEHT boost showed comparable results with conventional hyperthermia; potential therapeutic effects were also observed. Moreover, mEHT could be considered a useful tool in combination treatment with radiotherapy owing to its low thermotoxicity and improved treatment compliance.

**Abstract:**

We evaluated the effect of 13.56 MHz modulated electro-hyperthermia (mEHT) boost in neoadjuvant treatment for cT3-4- or cN-positive rectal cancer. Sixty patients who completed the mEHT feasibility trial (ClinicalTrials.gov Identifier: NCT02546596) were analyzed. Whole pelvis radiotherapy of 40 Gy, mEHT boost twice a week during radiotherapy, and surgical resection 6–8 weeks following radiotherapy were performed. The median age was 59. The median follow-up period was 58 (6–85) months. Total/near total tumor regression was observed in 20 patients (33.3%), including nine cases of complete response. T- and N-downstaging was identified in 40 (66.6%) and 53 (88.3%) patients, respectively. The 5-year overall and disease-free survival were 94.0% and 77.1%, respectively. mEHT energy of ≥3800 kJ potentially increased the overall survival (*p* = 0.039). The ypN-stage and perineural invasion were possible significant factors in disease-free (*p* = 0.003 and *p* = 0.005, respectively) and distant metastasis-free (*p* = 0.011 and *p* = 0.034, respectively) survival. Tumor regression, resection margin status, and other molecular genetic factors showed no correlation with survival. Although a limited analysis of a small number of patients, mEHT was feasible considering long-term survival. A relatively low dose irradiation (40 Gy) plus mEHT setting could ensure comparable clinical outcomes with possible mEHT-related prognostic features.

## 1. Introduction

Considering neoadjuvant treatment for rectal cancer, hyperthermia boost to radiochemotherapy reportedly produces excellent local control results; however, the long-term survival effects have not been sufficiently proven [1]. Attention is focused on whether this limitation of hyperthermia could be overcome by 13.56 MHz-based modulated electro-hyperthermia (mEHT), which possesses a potential cell killing effect by means of specific immunogenic pathways in addition to the traditional thermal effect [2,3,4].

mEHT has been demonstrated to possess effects at an average temperature of <39 °C [5,6]. In our previous early feasibility report for rectal cancer treatment, we demonstrated an excellent lymph node response by mEHT boost and a complementary nature of mEHT to radiation, thereby exploring the possibility of radiation dose reduction in combination with mEHT [7]. This study aimed to determine the follow-up results, focusing on the long-term survival of patients who faithfully received mEHT while undergoing neoadjuvant treatment for rectal cancer.

## 2. Materials and Methods

### 2.1. Patients

**Figure 1:**
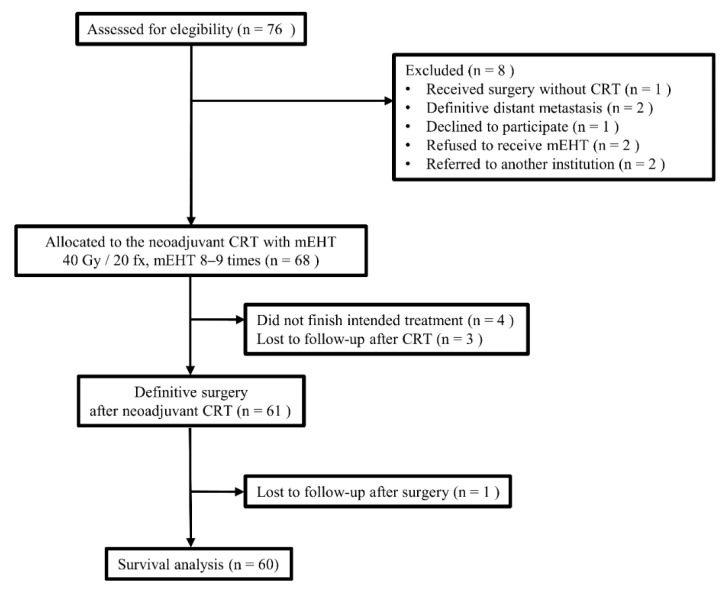
CONSORT diagram (CRT: chemoradiation, mEHT: modulated electro-hyperthermia).

This single non-inferior prospective trial received approval of the Institutional Review Board of Wonju Severance Christian Hospital (Approval number: CR313035) and was registered with ClinicalTrials.gov (study number NCT02546596), a total of 60 patients with cT3-4 or cN positive rectal cancer faithfully underwent preoperative radiochemotherapy with concomitant mEHT boost between March 2014 and March 2017 (Figure 1). For pre-treatment staging, magnetic resonance imaging and computed tomography were performed. All patients had a general condition of ECOG performance status ≤2. Considering the thermal toxicity, cases in whom we anticipated thermal hypersensitivity, such as severe cardiac conditions or excessive subcutaneous fat, were fundamentally excluded. The above have been described in detail in the previous early clinical feasibility report [7]. Patient- and disease-related characteristics are shown in Table 1.

### 2.2. Overall Treatment Schedule

Three- or four-field linear accelerator-based 6–15 MV X-rays from three-dimensional planning were delivered to the whole pelvis area including the rectal tumor, mesorectum, and internal iliac/presacral lymph node chain up to the sacral promontory level in 2 Gy daily fractions up to a total dose of 40 Gy. Intravenous 5-fluorouracil (400 mg/m^2^/day at the 1st and 5th weeks from the start of radiotherapy) or oral capecitabine (825 mg/m² based on the virtual period of the conventional 28-fraction radiation schedule) was administered concomitantly. According to the protocol, curative resection with lymph node dissection was planned at 6–8 weeks following completion of radiotherapy. Ultimately, the specific resection range was based on the surgeon’s discretion considering the tumor location, sphincter function, or clinical response to preoperative treatment.

### 2.3. Modulated Electro-Hyperthermia

In addition to chemoradiation, eight sessions of mEHT were combined twice weekly during the radiotherapy period using 13.56 MHz capacitive coupled device (EHY2000, Oncotherm GmbH, Troisdorf, Germany). Treatment was performed such that a 30 cm-diameter electrode included the entire treatment area based on the center of the irradiation site while the patient was in a supine position. Treatment duration per session was 60 min, and the interval between mEHT and radiotherapy on the same day was <1 h. The power to be applied was 140 W; only for the first session, a gradual power increase method (starting at 100 W to increase in 20 W per 20 min) was used in consideration of the patient’s adaptation status. In all subsequent sessions, the applied energy was partially adjusted when heat-related discomforts were recognized.

### 2.4. Treatment Response and Toxicities

Neoadjuvant treatment response and toxicity evaluation was performed as described in the previous early feasibility study [7,8]. The evaluation period of acute toxicity was from the start of neoadjuvant treatment to 90 days after ending radiotherapy, and toxic events that occurred thereafter were classified as late toxicity. Each toxicity grade during the period was based on the maximum value. Acute toxicity was assessed by NCI-CTCAE version 3.0 (NCI, Bethesda, MD, USA), while late toxicity was based on RTOG and EORTC criteria [9]. For the mEHT-related toxicity that mostly disappeared immediately after treatment, separate evaluation was conducted based on the Berlin scoring system, during the radiotherapy period only [10].

### 2.5. Statistical Analysis

In this study that assessed the follow-up results of the impact of mEHT boost on survival in a single-arm, non-inferiority trial, the primary endpoint (preoperative therapeutic response) assessment was by pathologic downstaging and tumor regression grade [7]. Survival rates were analyzed based on the baseline factors, in a median 58 (range, 6–85) months of follow-up. By definition, overall survival (OS) was the time interval from the day of radiotherapy to the day of death or last follow-up. Disease-free survival (DFS), loco-regional recurrence-free survival (LRRFS), and distant metastasis-free survival (DMFS) were determined to be from the day of surgery to the day of recurrence, death, or last follow-up, respectively. To examine the impact of each clinical parameter within a single group treated with radiotherapy plus mEHT, the difference in survival according to each parameter category was analyzed. Chi-square and Fisher’s exact tests were used for categorical variable analysis, as appropriate. Cox proportional hazard regression analysis was used to calculate univariate/multivariate adjusted hazard ratios (HRs) and 95% confidence intervals (CIs) for each survival. Statistical significance was based on *p* < 0.05. Analyses were conducted using SAS software 9.4 (SAS, Cary, NC, USA) and R 4.0.5 (Institute for Statistics and Mathematics, Vienna, Austria).

## 3. Results

### 3.1. Clinicopathology- and Treatment-Related Indices

**Figure 2:**
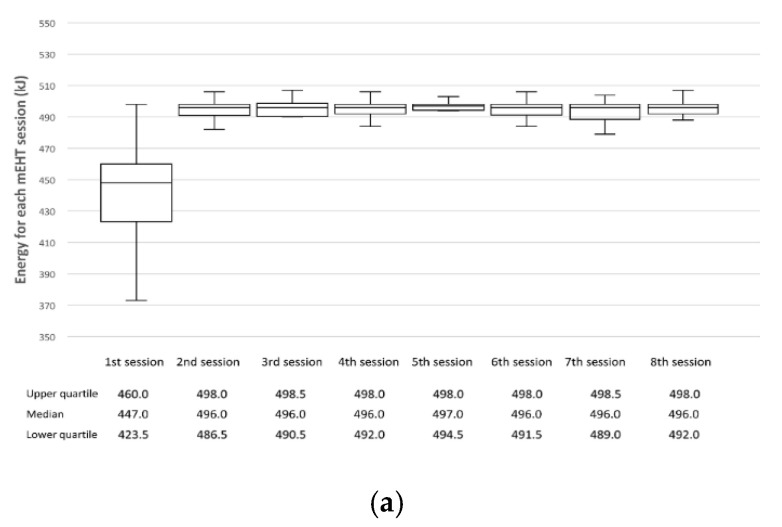

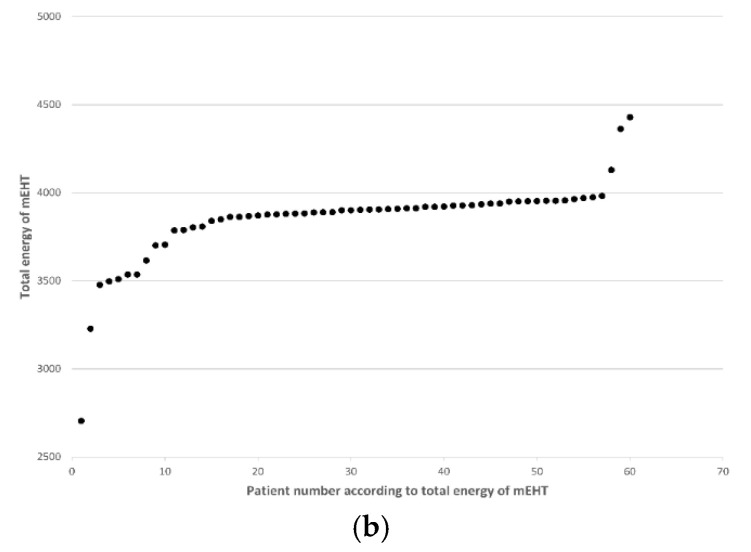
Energy profile of modulated electro-hyperthermia (mEHT) at each mEHT session (**a**) and from the perspective of total value line-up (**b**).

Factors that could affect the treatment outcomes, such as details of each treatment modality, response to preoperative treatment, and pathology after surgical resection, are shown in Table 2. The participants’ median age was 59 (range, 33–83) years, and they were predominantly male (*n* = 45, 75%). The clinical tumor volume had a median of 52.7 (range, 22.4–233.1) cm^3^. All patients completed their scheduled treatment course, including eight sessions of mEHT, whose median total energy was 3902 (range, 2704–4429) kJ (the energy value up to 8 sessions is shown in Figure 2a). At surgery, R0 resection was performed in 53 patients (88.3%); R2 resection was not performed. The proportion of lower ypT-stage (ypT0–2) and N-stage (ypN0) was 55.0% (33 patients) and 76.7% (46 patients), respectively. The number of relatively good treatment responses among patients (total and near total regression grade for primary tumors) was 20 (33.3%). All acute toxicity occurred within grade 2. As for late toxicity, there were no >grade 2 events other than grade 3 gastrointestinal toxicity in four cases. Among the analyzed patients, mEHT-related toxicity was mild in all but one grade 2 case (Table 3).

### 3.2. Survival

**Figure 3:**
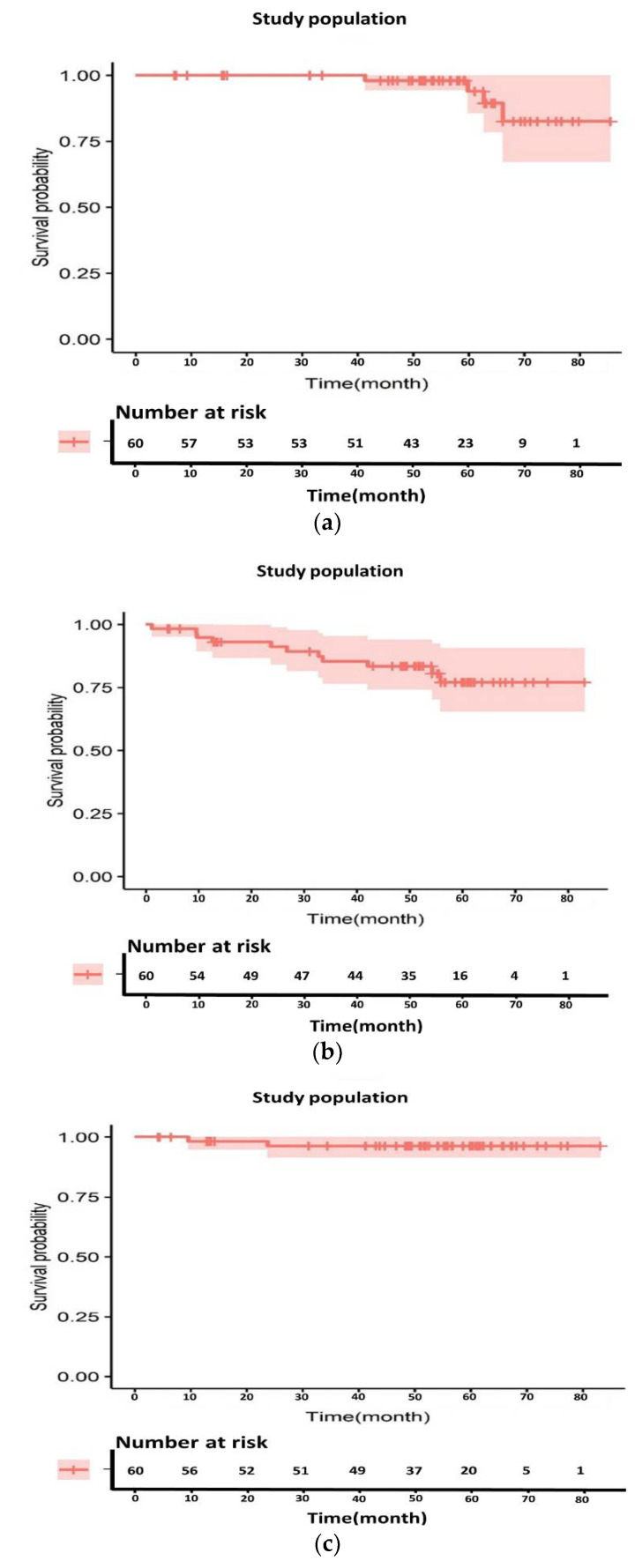

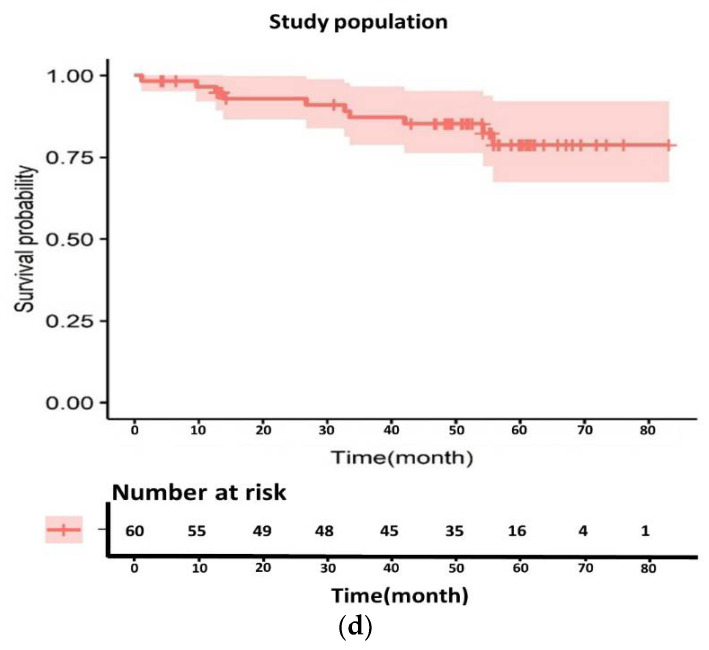
Survival analysis of the study population. (**a**) Overall survival, (**b**) disease-free survival, (**c**) loco-regional recurrence-free survival, and (**d**) distant metastasis-free survival.

**Figure 4:**
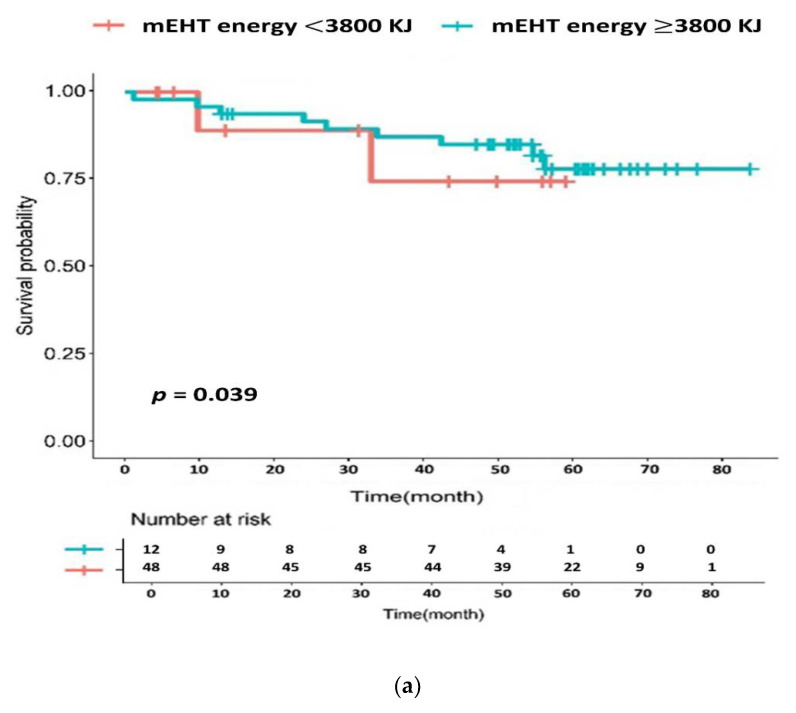

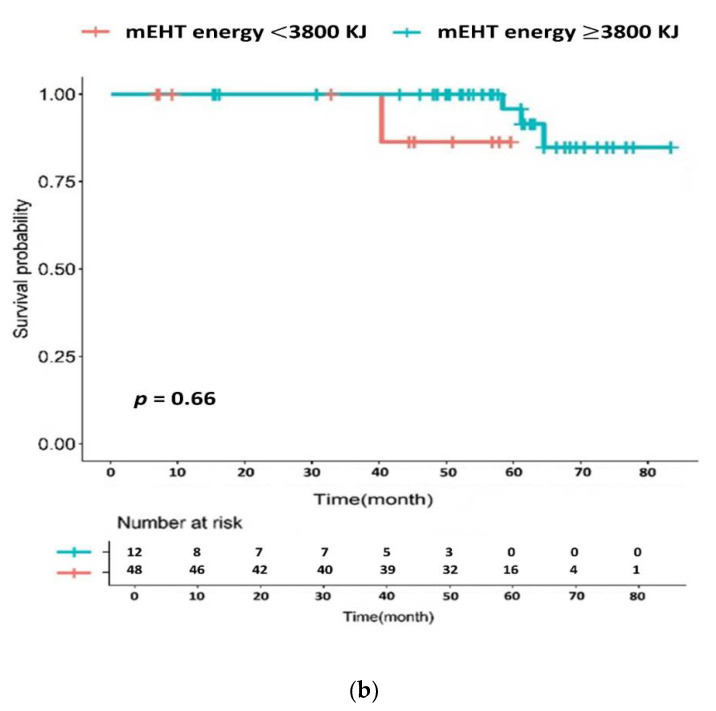
Survival comparison according to modulated electro-hyperthermia (mEHT) total energy by log rank test. (**a**) Overall survival, (**b**) disease-free survival.

**Figure 5:**
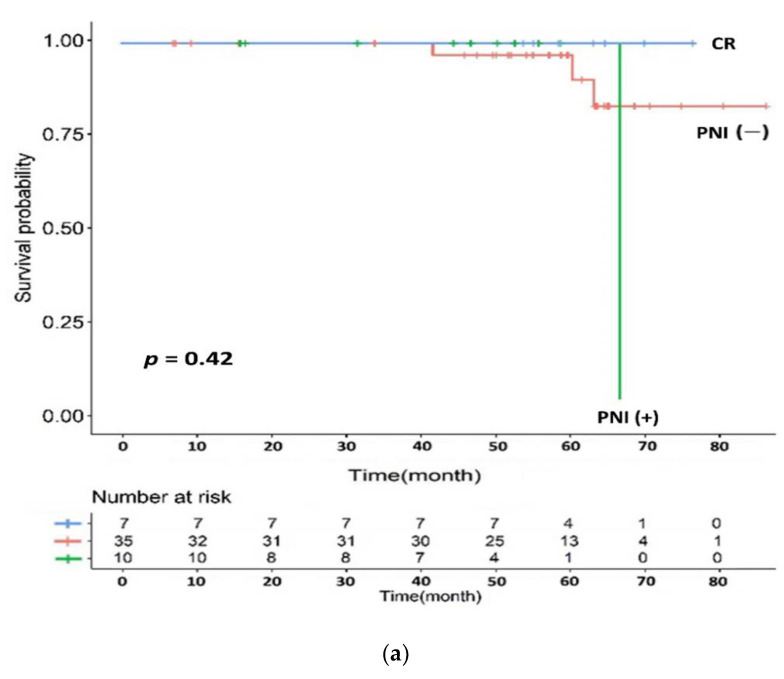

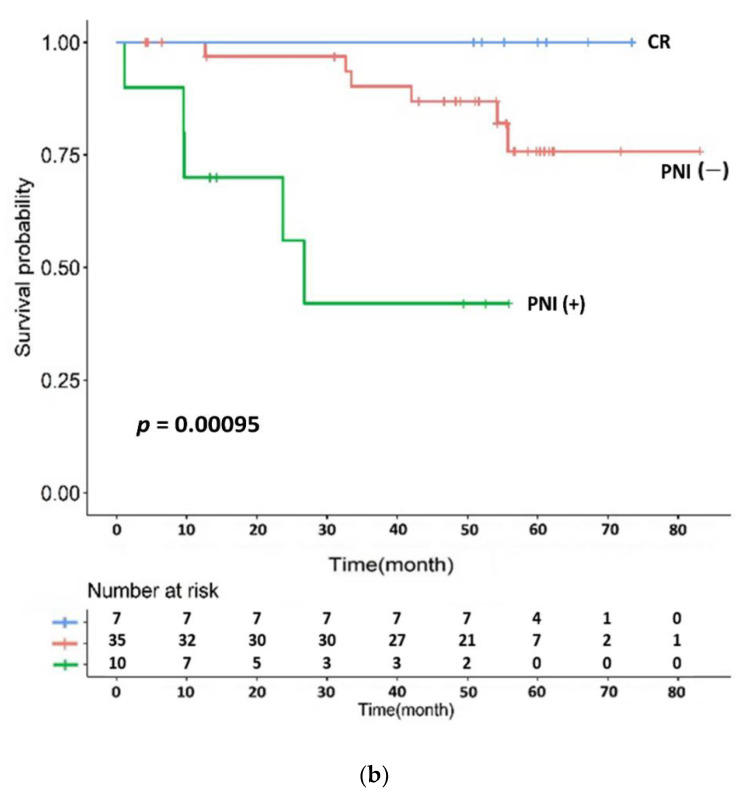
Survival comparison according to perineural invasion by log rank test. (**a**) Overall survival, (**b**) disease-free survival (CR: complete response, PNI: perineural invasion).

We included 60 patients for the log rank test and 52 patients for univariate/multivariate analysis, considering postoperative follow-up loss or missing values for various clinical factors. The 5-year OS, DFS, LRRFS, and DMFS rates were 94.0%, 77.1%, 96.4%, and 78.7%, respectively (Figure 3). A total of two loco-regional recurrences and 10 distant metastases occurred during the follow-up. Each recurrence site was the primary lesion (two patients) and peripheral lymph nodes (one patient), and one case of multiple recurrence or metastasis was observed. Two patients with loco-regional recurrence belonged to the low tumor regression group, one of whom had postoperative positive resection margin status. The distribution of the total mEHT energy showed a relatively rapid change around 3800 kJ, which was used as a cut-off value for the comparison of applied energy judged to be meaningful in terms of the relative balance between energy value categories except for the few extreme values (Figure 2b). When comparing 3800 kJ as a boundary, mEHT energy possibly affected the OS (Figure 4a). Differences according to molecular pathological factors, such as KRAS, BRAF, and microsatellite status, did not appear to affect survival in the mEHT-based group (Table 4). ypN-stage and perineural invasion (PNI) seemed to be related to DFS (*p* = 0.003 and *p* = 0.005, respectively for univariate analysis) and DMFS (*p* = 0.011 and *p* = 0.034, repectively for univariate analysis), which was more remarkable with the complete response group added (Figure 5b). Tumor regression and resection margin status, which are considered to be prognostic factors in preoperative chemoradiation, did not show significant correlation in our mEHT-based patient group (Table 4).

## 4. Discussion

In a recent retrospective analysis based on whether or not mEHT was supplemented, mEHT was effective in downstaging and tumor regression, which was more pronounced in large-sized tumors [11]. We attempted to assess how each clinical parameter affects the survival rates when mEHT is concurrently combined with radiation. This study was limited to ascertaining the significance of mEHT as it focused on descriptive data without a control group. Nevertheless, compared to previous studies on similar platforms, generally non-inferior survival outcomes were obtained. Although the patient characteristics were not completely consistent, 5-year OS and DFS of 94.0% and 77.1%, respectively, were similar to the results of survival improvement by conventional hyperthermia boost (Table 5). Generally, the addition of hyperthermia had excellent results for loco-regional control; however, it rarely resulted in an improvement in the survival rate [1,12].

Although mEHT-mediated survival gain was not clearly identified with a single-arm study, our non-inferior results at least demonstrated the usefulness of mEHT to some extent in the low radiation dose setting of 40 Gy. Despite attempts to improve the oncologic outcome through treatment intensification during chemoradiation, toxicity risk-related uncertainty still remains [13,14]. mEHT, which is relatively free from toxicity, is thought to be effective in more stable thermoradiotherapy. In addition, though very limited, the manageability of mEHT was revealed based on the concept of applied energy rather than intratumoral temperature without invasive parameter measurement. Recent mEHT studies have also reported good clinical cases regardless of temperature measurement [15,16]. Therefore, it is appropriate to investigate whether mEHT boost is a trigger for improving the clinical outcome through non-thermal effects, such as changes in the tumor microenvironment or immunogenicity, while being less affected by temperature.

Although limited, mEHT demonstrated the potential for survival improvement by increasing the total applied energy (Figure 4a). Unlike other previous clinical reports of hyperthermia, our study showed almost no variation in the mEHT-related parameters between patients as most patients possessed high treatment compliance and relatively uniform energy input above a certain level. Hence, the tendency in OS difference by energy level came from a structure wherein determining the prognosis was challenging owing to the tight energy distribution. Therefore, this result is thought to have its own clinical impact compared to the value obtained statistically, representing the importance of the input energy itself.

The low thermotoxicity of mEHT and its high therapeutic compliance are advantageous in terms of treatment management, including applied energy assessment. As originally planned, mEHT was performed in all patients twice a week. The energy for each session showed a slight increase generally up to the 8th session, which is directly contrary to common hyperthermia protocols (Figure 2a). Although the treatment compliance has been improving in conventional hyperthermia via technological advances [14], in most rectal cancer hyperthermia studies, the session number was insufficiently set to less than once a week or did not meet the schedule owing to thermotoxicity [10,12,17,18,19,20]. Therefore, mEHT application to the pelvic area is reportedly less associated with thermal toxicity, indicating that thermosensitive patients can adapt to the high-frequency energy as the session is repeated. The unexpectedly high heat sensitivity that appeared in some patients should be compensated by a more individualized approach. Another limitation of our study was that cases of severe obesity were excluded without clear criteria for heat sensitivity. If these cases are supplemented, discovery of biomarkers for mEHT indications and easier treatment application could be achieved.

Among molecular pathological factors, it was found that only PNI specifically affected the survival rates. PNI has been studied in several malignancies, including uterine cervical and head and neck cancers [21,22]; however, it has not been widely assessed in colorectal cancer. There have been limited reports in some colorectal cancer studies that PNI positivity could serve as a factor that lowers the survival rates [23,24,25]. Thus, a more in-depth study of PNI is needed in terms of the specific situation of mEHT-based neoadjuvant thermoradiotherapy.

In a previous retrospective analysis that included a control group (non-mEHT group), the resection margin status was one of the significant prognostic factors for survival [11]; however, this trend disappeared in this mEHT-dominant group. This could be the result of the difference in the follow-up period or the relatively small number of patients. However, the mEHT-mediated impact also needs to be confirmed, i.e., whether it is large enough to offset the influence of the resection margin, etc. Meanwhile, besides the role of mEHT, it is worth noting that 40 Gy radiation may be sufficient for the neoadjuvant treatment for rectal cancer, which is consistent with the latest report that 40–41.4 Gy was sufficient for esophageal cancer treatment [26,27]. Nevertheless, an index comparable to intratumoral temperature has not been established, which is a contemporary problem that needs to be continuously addressed in terms of the quality management of mEHT. These limitations in this study will have to be overcome through a large-scale prospective well-designed clinical trial in the future.

**Table 5:** Comparison of overall and disease-free survival in previous neoadjuvant thermoradiotherapy studies for rectal cancer.

References	Patient Enrollment	No. of Patients	Radiation Dose	Hyperthermia Machine	No. of Hyperthermia Session	Overall Survival	Disease-Free Survival
Maluta et al., 2010 [18]	Phase II	76	60 Gy (50 Gy + 10 Gy boost)/30 times	BSD-2000	Once a week (5 times)	86.5% (5 years)	74.5% (5 years)
Kang et al., 2011 [12]	Retrospective	98	Group A: 39.6 Gy /22 times, Group B: 45.0 Gy/25 times	Cancermia GHT-RF8	Twice a week (1–11 times)	73.9% (5 years)	75.1% (5 years)
Gani et al., 2016 [28]	Retrospective	60	50.4 Gy/28 times	BSD-2000	once or twicea week (1–9 times)	88.0% (5 years)	77.0% (5 years)
Gani et al., 2021 [29]	Phase II	78	50.4 Gy/28 times	BSD-2000	Twice a week (1–10 times)	94.0% (3 years)	81.0% (3 years)
Ott et al., 2021 [14]	Prospective	89	50.4 Gy/28 times	BSD-2000	Twice a week (1–11 times)	82.0% (5 years)	57.0% (5 years)
Current study	Phase II	60	40 Gy/20 times	Oncothermia EHY-2000	Twice a week (8–9 times)	94.0% (5 years)	77.1% (5 years)

## 5. Conclusions

A non-inferior effect of 40 Gy radiation plus mEHT combination was substantiated in the long-term survival of patients. In a slightly low-dose radiation platform, less thermotoxic mEHT can be considered to aid in rectal cancer treatment. In the long term, a segregated approach from conventional hyperthermia is warranted in the overall management with a reasonable consensus on the applied energy index.

## Figures and Tables

**Table 1:** Patient- and disease-related characteristics at diagnosis (*n* = 60).

Characteristic		Value
Age (year)	<60 ≥60	32 (53.3%) 28 (46.7%)
Sex	Male Female	45 (75.0%) 15 (25.0%)
Pathologic diagnosis	Adenocarcinoma Mucinous adenocarcinoma Tubular adenocarcinoma	57 (95.0%) 2 (3.3%) 1 (1.7%)
Histological differentiation	Well-differentiated Moderately differentiated Poorly differentiated	8 (13.3%) 49 (81.7%) 3 (5.0%)
Primary tumor location from the anal verge (cm)	≤5 >5	23 (38.3%) 37 (61.7%)
Primary tumor volume (cm^3^)	<65 ≥65	41 (68.3%) 19 (31.7%)
Positive lymph node volume (cm^3^)	≤5 >5	34 (55.0%) 27 (45.0%)
cT stage	T3 T4	46 (76.7%) 14 (23.3%)
cN stage	N1 N2	28 (46.7%) 32 (53.3%)
Carcinoembryonic antigen (ng/mL)	≤5 >5	39 (65.0%) 21 (35.0%)
Carbohydrate antigen 19–9 (U/mL)	≤37 >37 Not available	50 (83.3%) 9 (15.0%) 1 (1.7%)
KRAS mutation	Negative Positive Not available	27 (45.0%) 14 (23.3%) 19 (31.7%)
BRAF mutation	Negative Positive Not available	38 (63.3%) 2 (3.3%) 20 (33.3)
Microsatellite instability	Microsatellite-stable Microsatellite instability-low Microsatellite instability-high Not available	38 (63.3%) 1 (1.7%) 1 (1.7%) 20 (33.3%)

**Table 2:** Factors associated with neoadjuvant treatment and surgical outcomes (*n* = 60).

Characteristic		Value
Total dose of radiotherapy		40 Gy
Total number of mEHT session		Median 8 (range, 8–9)
Total energy of mEHT (kJ)	<3800 ≥3800	12 (20.0%) 48 (80.0%)
Chemotherapy regimen	5-fluorouracil/leucovorin Capecitabine Others	4 (6.7%) 55 (91.7%) 1 (1.7%)
Radiotherapy to surgery interval (day)		Median 52 (range, 41–70)
Types of Surgery	Low anterior resection Abdominoperineal resection Hartmann’s procedure Others	50 (83.3%) 4 (6.7%) 3 (5.0%) 3 (5.0%)
Resection margin status	Negative Positive	53 (88.3%) 7 (11.7%)
ypT	CR, Tis, T1, T2 T3, T4	33 (55.0%) 27 (45.0%)
ypN	N0 N1, N2	46 (76.7%) 14 (23.3%)
Stage group	CR, 0(TisN0), I, II, III	26 (43.3%) 34 (56.7%)
Tumor regression grade	Total, near total Moderate, minimal	20 (33.3%) 40 (66.7%)
Lymphatic invasion	Negative Positive Complete response Not available	41 (68.3%) 5 (8.3%) 9 (15.0%) 5 (8.3%)
Venous invasion	Negative Positive Complete response Not available	43 (71.7%) 3 (5.0%) 9 (15.0%) 5 (8.3%)
Perineural invasion	Negative Positive Complete response Not available	36 (60.0%) 10 (16.7%) 9 (15.0%) 5 (8.3%)
Tumor budding	Negative Positive Complete response Not available	16 (26.7%) 26 (43.3%) 9 (15.0%) 9 (15.0%)

mEHT: modulated electro-hyperthermia, SD: standard deviation, CR: complete response.

**Table 3:** Distribution of treatment-related toxicities (*n* = 60).

Toxicity Grade	0	1	2	3	4	5	NA
Acute GI	20	21	19	0	0	0	0
Acute GU	47	12	1	0	0	0	0
mEHT-related *	44	15	1	0	0	0	0
Late GI	16	16	15	4	0	0	9
Late GU	41	11	3	0	0	0	5

GI: gastrointestinal. GU: genitourinary. mEHT: modulated electro-hyperthermia. NA: not available. * Scoring system proposed by the Berlin group [10].

**Table 4:** Univariate and multivariate analyses of the baseline variables.

Variable	Category	Univariate			Multivariate		
		HR	95% CI	*p*	HR	95% CI	*p*
Overall Survival							
Age (years)	<60 vs. ≥60	1.318	0.184–9.433	0.783	2.990	0.201–44.527	0.429
Sex	Male vs. Female	1.399	0.143–13.695	0.773	2.468	0.164–37.189	0.514
Resection margin status	Negative vs. Positive	9.200	0.575–147.73	0.117	59.458	0.150–23546.9	0.181
ypN-stage	0 vs. 1, 2	1.042	0.104–10.480	0.972	2.111	0.084–53.349	0.650
Tumor regression grade	Total, near total vs. Moderate, minimal	0.574	0.079–4.188	0.584	0.111	0.003–4.608	0.248
Total mEHT energy (kJ)	<3800 vs. ≥3800	0.103	0.006–1.869	0.124	0.402	0.008–19.397	0.645
Disease-free Survival							
Age (years)	<60 vs. ≥60	1.005	0.306–3.297	0.993	1.503	0.386–5.849	0.557
Sex	Male vs. Female	1.061	0.281–4.007	0.930	2.093	0.505–8.669	0.308
Resection margin status	Negative vs. Positive	2.057	0.442–9.568	0.358	5.623	0.375–84.259	0.211
ypN-stage	0 vs. 1, 2	6.630	1.916–22.934	0.003	5.831	0.955–35.594	0.056
Tumor regression grade	Total, near total vs. Moderate, minimal	1.538	0.407–5.811	0.526	0.223	0.036–1.396	0.109
Perineural invasion	Negative vs. Positive	5.744	1.687–19.559	0.005	4.487	0.818–24.630	0.084
Total mEHT energy (kJ)	<3800 vs. ≥3800	0.866	0.186–4.037	0.855	0.311	0.311–49.627	0.290
Loco-regional Recurrence-free Survival							
Age (years)	<60 vs. ≥60	1.239	0.077–19.802	0.880	5.232	0.078–349.23	0.440
Sex	Male vs. Female	2.622	0.164–41.953	0.496	7.443	0.185–298.65	0.287
Resection margin status	Negative vs. Positive	8.571	0.530–138.55	0.130	60.406	0.397–9196.8	0.110
ypN-stage	0 vs. 1, 2	3.087	0.193–49.355	0.425	5.937	0.305–115.46	0.240
Distant Metastasis-free Survival							
Age (years)	<60 vs. ≥60	0.793	0.224–2.811	0.719	0.928	0.229–3.758	0.917
Sex	Male vs. Female	1.208	0.311–4.687	0.784	2.093	0.498–8.793	0.313
Resection margin status	Negative vs. Positive	2.270	0.479–10.768	0.302	4.262	0.311–58.359	0.278
ypN-stage	0 vs. 1, 2	5.341	1.461–19.525	0.011	5.916	0.899–38.941	0.065
Tumor regression grade	Total, near total vs. Moderate, minimal	1.325	0.342–5.137	0.684	0.204	0.033–1.274	0.089
Perineural invasion	Negative vs. Positive	4.082	1.111–14.998	0.034	2.467	0.430–14.146	0.311
Total mEHT energy (kJ)	<3800 vs. ≥3800	0.737	0.155–3.498	0.701	2.221	0.219–22.515	0.500

HR: hazard ratio, CI: confidence interval, mEHT: modulated electro-hyperthermia.

## Data Availability

Data used in this study can be provided by the corresponding authors upon request. Data cannot be shared publicly due to privacy concerns.
